# Multiplex Immunofluorescence and Multispectral Imaging: Forming the Basis of a Clinical Test Platform for Immuno-Oncology

**DOI:** 10.3389/fmolb.2021.674747

**Published:** 2021-06-02

**Authors:** Clifford C. Hoyt

**Affiliations:** ^1^ Akoya Biosciences Inc., Malborough, MA, United States

**Keywords:** Predictive biomarkers, multiplex immunofluorescence, multispectral imaging, immuno-oncology, clinical workflow, image analysis, automated staining, spatial biology

## Abstract

As immuno-oncology (I/O) emerges as an effective approach in the fight against cancer, multispectral imaging of multiplex immunofluorescence (mIF) is maturing as an analytical platform. The timing is fortuitous. Due to health economic considerations surrounding the use of I/O, there is an urgent need for tests that accurately predict response to the growing list of available therapies. Multispectral mIF provides several advantages over other biomarker modalities by enabling deeper interrogation of the intricate biology within the tumor microenvironment, including detection of cell-to-cell spatial interactions that correlate with clinical outcomes. It also provides a practical path for generating reliable and reproducible results in a clinically suitable, high-throughput workflow. In this article, we (1) describe the principles behind multispectral mIF; (2) provide advice and recommendations on assay development and optimization and highlight characteristics of a well-performing assay; and (3) discuss the requirements for translating this approach into clinical practice.

## Introduction

Tissue biopsies and surgical resections offer a critical insight into a patient’s cancer and are the basis of prognostic evaluations and therapy selection. Yet a wealth of information remains largely inaccessible due to the limitations of established tools and methods for evaluating formalin-fixed, paraffin-embedded (FFPE) tissue sections. For example, immunohistochemistry (IHC), the established tool for characterizing the biology present in the tumor and its microenvironment, lacks the capability to capture the complexity of cell-to-cell biology because it reveals only one or two proteins at a time. Pathologist assessment of tissues is done primarily by visual inspection and includes review of the tissue morphology, and positivity of one or two proteins, or of two to three genes targeting DNA or RNA molecules. Its application to assessments of expression in cellular subgroups is also inconsistent because it relies solely on visual interpretation which may vary greatly between individuals (Hirsch et al., 2017; Ilie and Hofman, 2017; Rimm et al., 2017).

Recent advances in tissue image analysis are helping address the variability and limitations of human perception. Measuring biologically significant parameters that are substantially out of reach of human perception, such as cellular co-expression, cellular spatial relationships, tissue heterogeneity, and expression of low abundance molecules, is now possible. However, progress to date with the application of quantitative image analysis, including deep learning and artificial intelligence approaches, has focused mainly on conventional IHC and hematoxylin and eosin (H&E)–stained slides, which does not leverage the wealth of data that can be captured through multiplex immunofluorescence (mIF) (Effner et al., 2019; Niazi et al., 2019).

Understanding the cellular composition and spatial distribution in tissue sections, termed “spatial biology,” is particularly valuable in the age of immunotherapy. Immune checkpoint inhibitors have revolutionized cancer treatment, especially for metastatic disease, for which patients have little recourse. Immune checkpoint inhibitors reduce T-cell inhibition, allowing them to attack cancers unhindered. In instances where a patient positively responds to the therapy, the benefits often last for years rather than months, which has led to excitement for potential cures for cancer (Wilky, 2019).

While lifesaving for some, current I/O treatments offer little benefit to more than 80% of patients (Haslam and Prasad, 2019; Wilky, 2019). With costs typically twice that of other types of cancer treatments, with frequent and impactful side effects, and with often precious little time for metastatic patients to try different approaches, there is an urgent need for better predictive assays that can be used to determine which drug or combination of treatments is most likely to help (Mehnert et al., 2017). This is particularly important with the rapid increase in the number of trials involving combination therapy approaches. Combination therapies target multiple proteins and/or cellular signaling cascades to provide more impactful treatment. Clinical trials have not only shown that patients can have significantly higher response rates but have also shown higher frequency of severe side effects. Predictive tests to help oncologists identify likelihood of response for monotherapy vs. combination therapy will have significant health economic benefits.

However, predicting whether a patient will respond or not to immunotherapies has proven difficult. This is probably due to how complex cancer is and how it uses multiple mechanisms to evade the immune system and survive. Currently, few approved diagnostic approaches exist that can accurately determine the likelihood of response to the ever-expanding list of FDA-approved immunotherapy drugs.

There is mounting evidence that the spatial biology occurring within the tumor microenvironment (TME) holds the answers as to why some patients respond to immunotherapy and others do not. Early in the I/O era, Tumeh et al. used quantitative IHC and mIF to investigate advanced melanoma patient responsiveness to pembrolizumab. They found that the presence of CD8^+^ cytotoxic T-cells present along the invasive margin of the tumor, as well as the close proximity of programmed death receptor 1 (PD-1, located on CD8^+^ T-cells) to programmed death ligand 1 (PD-L1), predicted the therapeutic response to anti–PD-1 blockade and subsequent tumor regression (Tumeh et al., 2014). In another study, Johnson et al. demonstrated a similar finding in melanoma based on PD-1 and PD-L1 proximity to other cell types within the TME (Johnson et al., 2016). Since then, there have been several biomarker studies performed that highlight the performance of mIF and the value of spatial biology (Gettinger et al., 2018; Giraldo et al., 2018; Mazzaschi et al., 2018; Wong et al., 2018; Althammer et al., 2019).

An interdisciplinary team led by Johns Hopkins University recently conducted a meta-analysis on data pooled from more than 50 studies, spanning more than 10 cancer types and over 8,000 patients (Lu et al., 2019). Each study assessed the predictive value of one or more biomarker assays intended to determine the likelihood of response to anti–PD-1/PD-L1 therapy, the leading class of immunotherapy. The meta-analysis revealed that three of the four assays most commonly utilized in I/O research, PD-L1 IHC, tumor mutation burden (TMB), and gene expression profiling (GEP), had moderate, comparable performance when predicting response to anti–PD-1/PD-L1 therapy. Interestingly, it revealed that the category of multiplex IHC or immunofluorescence (IF), which includes multispectral mIF, performed significantly better than the other three assay types.

The researchers concluded that the spatial biology revealed by mIHC or mIF, including cellular protein co-expression, localization, and arrangement, correlated better with patient response than information gathered with the other approaches. These findings support the premise that determining or predicting a patient’s likelihood to respond to a specific therapy will be aided by detailed cell-level evaluation of the TME-specific cell presence, their functional status, and how they interact within the TME.

**FIGURE 1:**
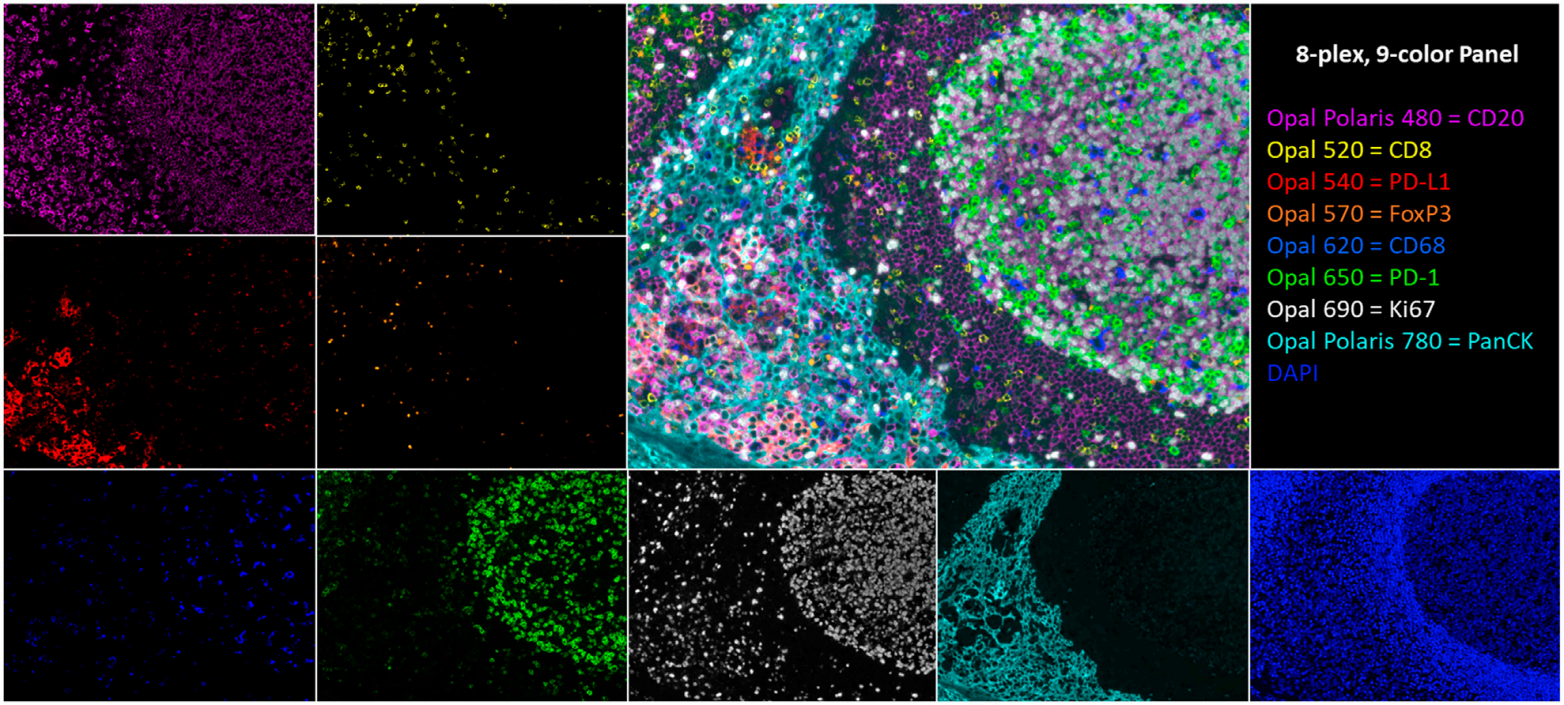
An 8-plex, 9-color tonsil composite image with respective channel monoplex images: Opal Polaris 480 = CD20 (purple), Opal 520 = CD8 (yellow), Opal 540 = PD-L1 (red), Opal 570 = FoxP3 (orange), Opal 620 = CD68 (blue), Opal 650 = PD-1 (green), Opal 690 = Ki67 (white), Opal Polaris 780 = PanCK (teal), and DAPI counterstain.

Ideally, to satisfy the urgent need for predictive I/O biomarkers, one would want to leverage the well-established attributes and benefits of conventional IHC and IF while taking advantage of new technologies for multiplex staining, high-throughput slide imaging, and computer vision, to provide an automated, reliable, and practical analysis workflow. To that end, we developed the multispectral mIF platform described here using a range of technologies to achieve an assay that is rapid, reproducible, and customizable to support research, clinical trials, and eventually standard of care. The platform consists of automated mIF staining using tyramide signal amplification (TSA), high-throughput multispectral slide image acquisition, and advanced machine learning–based image analysis algorithms for segmenting and characterizing the cell-level immuno-biology occurring in the TME (Stack et al., 2014). Example imagery is shown in Figure 1.

The goal of this article was to provide (1) the principles behind multispectral mIF; (2) high-level guidance on assay optimization to achieve sensitive reproducible multiplex assays; (3) performance metrics that typify well-optimized assays; and (4) a list of considerations for translating this method and methods like it into clinical standard of care. The perspectives and recommendations provided within this article are based on our experience with the Akoya mIF platform. We are sharing with the expectation that they might be useful generally for anyone developing mIF assays for translational work and eventual clinical use.

## Principles of Multispectral mIF

Before discussing the principles behind multispectral mIF, we will discuss assay performance goals as they drive the selection of the necessary technologies to perform the assay.

### Assay Performance Goals

Multiplex immunofluorescence methods have become a standard in I/O research, to understand cell-level biology occurring in the TME. However, little standardization has occurred. Up until now, IF and in situ hybridization approaches have been generally used to create imagery for qualitative visual assessment or to be analyzed ad hoc in the research setting with image analysis software packages designed to be open and flexible to suite individual project goals.

To advance quantitative mIF forward to support translational research and eventual clinical use, analytical performance standards are required at a level suitable for cancer diagnostic testing where accurate detection of cell types and biology depends on reliable detection of multiple proteins. The goal, effectively, is to create a quantitative multiplex imaging enzyme-linked immunosorbent assay (ELISA). Each pixel in an image of multiplex-stained samples should hold calibrated and precise data that indicate relative abundance of the multiple proteins of interest. If ELISAs are the analytical gold standard of protein measurement, the goal is to have percent co-efficient of variation (CV) of approximately 10% for detecting truly positive cells for one or more markers (Gupta et al., 2017). Analytical performance at this level is needed to support the following: (1) discovery of biomarkers that are real and reproducible; (2) the quality, regulatory, and analytical requirements for research studies and clinical trials; and (3) practical and reliable clinical deployment.

### Principles

The first of three guiding principles of current multispectral mIF, thanks to valuable insights provided by collaborators at leading academic medical institutions, is that the mIF assay matches the sensitivity of highly optimized conventional chromogenic IHC staining (Kim et al., 2016; Parra et al., 2017). Conventional IHC is the prevailing clinical standard and has served the medical community well. One might think initially that replicating conventional DAB staining sensitivity with a fluorescence assay would be straightforward; however, it poses technical challenges. Conventional IHC is often saturated to reveal weak expressing cells, driven by clinical evidence that low-level expression correlates with response (Caruana et al., 2020). Saturated DAB staining supports how a pathologist would visually assess a sample because it reveals both high- and low-level expressing cells, which is what the pathologist cares about. Visual acuity is also adept at distinguishing specific staining in the presence of diffuse nonspecific background staining. Visual analysis combined with IHC presents a very sensitive method for detecting positive cells, wherein positivity is determined using visual acuity to detect specific staining above background staining.

However, as mentioned earlier, visual assessment of conventional IHC is predominantly limited by multiplexing level and the subjectivity of human perception (Sapino et al., 2013; Troncone and Gridelli, 2017; Santana MFCdLF, 2018). When transitioning to a quantitative mIF assay, analytical performance needs to support all of the attributes of fluorescence detection compared to chromogenic, including quantitative measure of expression through linear dynamic range, and independent staining of each marker to support accurate and reproducible machine vision–based assessments of cellular co-expression, arrangement, and localization within the TME architecture and across whole sections.

The second guiding principle is that multispectral mIF staining needs to support consistent and accurate image analysis along with practical and fast process workflows. Analytical performance of a multispectral mIF assay depends on the integration of panel design and image analysis algorithms. While imagery can be visually enticing because of the biology it reveals, reliable and specific biomarker signatures are needed for translation. They form the basis of scores that will be used to make critical drug trial, and eventual clinical, decisions once appropriate regulatory certifications are obtained.

To this end, it is advantageous to include markers in multiplex assay panels that support image analysis functions to segment tissues and cellular compartments. This is critical given the variability of human tissues. For example, a tumor-specific marker or cocktail is essential to segment tumor regions and separate them from stroma. Also, if multiplexing bandwidth affords, including a cocktail of markers to serve as a “membrane counterstain” supports more robust cell segmentation by revealing cell surfaces to assist with assigning measured signals accurately to individual cells and with cell splitting, which can be challenging in tertiary lymphoid structures.

Another important attribute of robust assays is that they have strong and stable fluorescence signals to support rapid slide scanning and subsequent rescanning if needed, which may be needed months later. Stability includes two considerations—photostability to avoid bleaching from strong excitation light and stability of slides in storage. Two other attributes of robust assays are low background and independence of individual stains. These support accurate identification of cells-of-interest, frequency of colocalized markers, percent positivity, determination specific cell types within tissue microenvironments, proximities between certain cells, and other cellular distribution measures.

The third guiding principle is that the assay workflow needs to be practical, economical, and aligned with study and research laboratory standards so that this method is accessible to the entire research community, to accelerate and increase the likelihood of finding the most effective biomarkers. Furthermore, having a workflow that fits into clinical laboratory standards and workflows supports (1) pathologists who will continue to play a critical quality control and data review role, (2) laboratory and clinical personnel who will run the assays, and (3) physicians who need reliable and actionable information with rapid turnaround times.

## Technical Approach

To achieve these goals, we developed an end-to-end workflow based on the Akoya mIF platform that includes reagents for automated and manual staining (antibodies and detection reagents), image acquisition instruments capable of both field-of-view and multispectral whole-slide imaging, software applications for image analysis, data reduction, and a cloud-based image storage, sharing, and viewing solution. In the workflow described here, we used the Leica BOND RX autostainer for automated staining. Additional R-script packages help consolidate field-of-view datasets and investigate whole-slide parameters that support the research and clinical trial objectives of today (Stack et al., 2014). Developing an effective workflow requires careful and seamless integration of each individual component. Assay panels are designed and optimized to work with the image acquisition instruments and image analysis programs. The imaging instrument is configured to isolate and measure signals, which are spatially and spectrally overlapping. Lastly, the image analysis software is built from the ground up to support multispectral unmixing and tissue and cell segmentation based on specific staining patterns, with algorithms for cell phenotyping and expression thresholding, that are robust across the variability of human diseased tissue types.

This approach was selected as a focus for translational, and eventual clinical, work rather than other higher-plex options, including Akoya’s CODEX platform, other cycled mIF technologies, and approaches based on imaging mass spectrometry, because these platforms have attributes that would make it challenging to advance discovered biomarkers into a suitable clinical workflow. Contributing attributes that would make other approaches challenging are throughput, cost, and, in most cases, sensitivity which is needed to capture the intricate biology occurring in the TME related to therapy response.

Other mIF staining technologies, such as those from Ultivue, offer a single component of an end-to-end solution that could support translational work and eventual clinical application. The approach we describe in this review offers integration of a complete workflow which is important for assay reproducibility, accuracy, and standardization as each component of the workflow is optimized to support the other components. It also offers flexibility to discover and validate signatures among an almost infinite array of biological mechanisms to explore.

**FIGURE 2:**
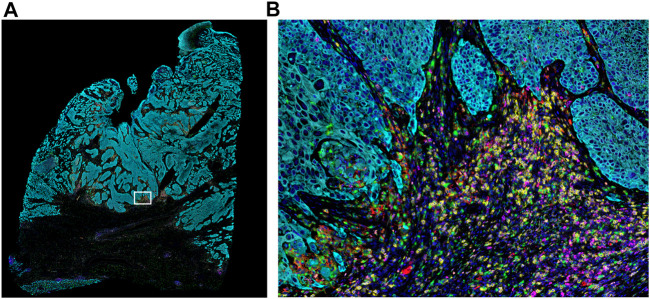
Whole-slide MOTiF image of lung cancer FFPE tissue. **(A)** Markers stained for this 6-plex, 7-color assay include CD8 (yellow), PD-L1 (red), FoxP3 (orange), PD-1 (magenta), cytokeratin (cyan), and CD68 (green). White box indicates **(B)** selected area from whole tissue section image at 20× magnification highlighting the interactions between the immune system and the tumor (i.e., “hotspot”). The cellular composition and distribution reveal immune engagement with the tumor, evidenced by tumor-infiltrating lymphocytes (TILs), PD-L1+ macrophages, PD-L1− tumor cells, and an abundance of para-tumoral regulator T-cells and cytotoxic T-cells, including several that are PD-1+. Elucidating the interplay between these different cell types is key to understanding the variance in patient responsiveness to therapeutic treatments.

The multispectral mIF platform described here utilizes TSA to support biomarker detection. TSA is a technology invented more than 20 years ago that amplifies IF detection through the use of horse radish peroxidase (HRP) to enzymatically convert TSA molecules into free radicals that then covalently bind to tyrosine residues on and in the immediate vicinity of the protein epitope targeted by the primary antibody (Figure 2) (Bobrow et al., 2001). Today, TSA technology has been optimized for integration into the multispectral mIF platform and is available under the Opal trademark (www.akoyabio.com). This technology enables the detection of low-level expression by elevating signal above background tissue autofluorescence. TSA is also very photostable relative to conventional IF methods, enabling the storage and re-scanning of slides a year after slides are stained without appreciable loss of signal. As each color is amplified individually, signals can be balanced for measurement with negligible spectral channel-to-channel bleed-through.

The fluorophores selected for Opal detection support up to 8-plex staining (9-colors including DAPI counterstain) and have been carefully selected to provide optimum spectral separation across the visible wavelength range. Fluorophore selection was based on detailed models of total system spectral response covering the entire optical train of the imaging system.

Amplified detection signals enable rapid slide scanning rates, typically with camera exposure times in the millisecond range for each fluorophore. This signal level translates to slide scan times of approximately 15 min per 7-color assay at 20× performed on a typical resection biopsy with an area of 1.5 × 1.5 cm (0.5 × 0.5 micron pixel size).

Having adjustable amplification gives researchers the flexibility to tailor assays to characterize biological mechanisms of interest, which may be best assessed by either high amplification to detect weak expressing cells or optimization to measure a large dynamic of expression if different expression levels have biological meaning. For example, if detecting very low expression is important, one can amplify aggressively. On the contrary, if a user wanted to capture as many gray-scale levels as possible, including at the high end of expression, one could amplify less aggressively.

There is a common belief that TSA-based amplification leads to variability in measured signals. This may be due, in part, to users not fully understanding all of the important parameters that need to be optimized to assure consistent, reproducible results. Careful assay development and optimization leads to reproducible measured signals, as evidenced by over 200 peer-reviewed articles that utilize the AQUA technology (McCabe et al., 2005), one of the first demonstrations of quantitative mIF, and by the rapidly growing number of more than 100 peer-reviewed articles describing results using the Opal technology. Just recently, a six-center inter-site comparison study, termed the multi-institutional TSA-amplified mIF reproducibility evaluation (MITRE) study, was undertaken to demonstrate the reproducibility of this integrated workflow system. The results revealed that multispectral mIF is not only transferable among different sites, but it is also reproducible at a level comparable to that of quantitative ELISAs, with CVs of <15% (Hoyt et al., 2019).

The MITRE study utilized the multispectral mIF workflow described in this article, including staining automation using the Leica BOND RX autostainer. The BOND RX autostainer is capable of staining 30 slides in a single run. Each run takes approximately 12–13 h, which fits into a daily schedule that includes sample and instrument preparation during the day and slide staining at night.

**FIGURE 3:**
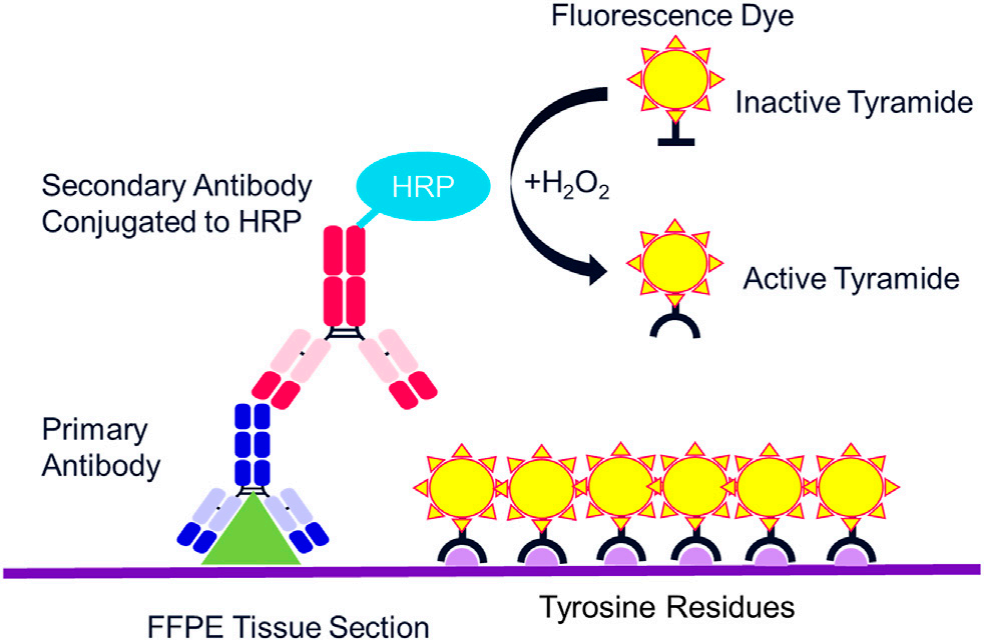
Mechanism of tyramide signal amplification (TSA) staining. Opal dyes allow for the use of any standard unlabeled primary antibody, including multiple antibodies raised in the same species. After introduction of the primary antibody, the Opal polymer HRP is applied. The Opal system uses TSA to amplify IHC detection by covalently depositing multiple fluorophores near that targeted antigen. After labeling is complete, antibodies are removed in a manner that does not disrupt the Opal fluorescence signal, allowing for the next target to be detected without antibody cross-reactivity.

Once the slides are stained, they are scanned on a multispectral digital slide imaging system, the Vectra Polaris. The Vectra Polaris uses patented multispectral imaging technology to compensate for optical spectral bleed-through among channels and to isolate signal from background autofluorescence, which is particularly important for fluorophores at the blue-to-green end of the visible spectrum (Figure 3). In an internal quantitative assessment investigating the ability of multispectral unmixing to compensate for spectral bleed-through, we found that the average optical bleed-through was 8.7% for an optimized 6-plex assay and 13% for an optimized 8-plex assay. With multispectral unmixing, residual bleed-through was reduced to <1% in both cases. If a signal is 10 times its spectral neighbor, a 10% bleed-through from the stronger channel into the weaker channel would be equivalent to the signal in the weaker channel, leading to significant false-positive cell classifications.

**FIGURE 4:**
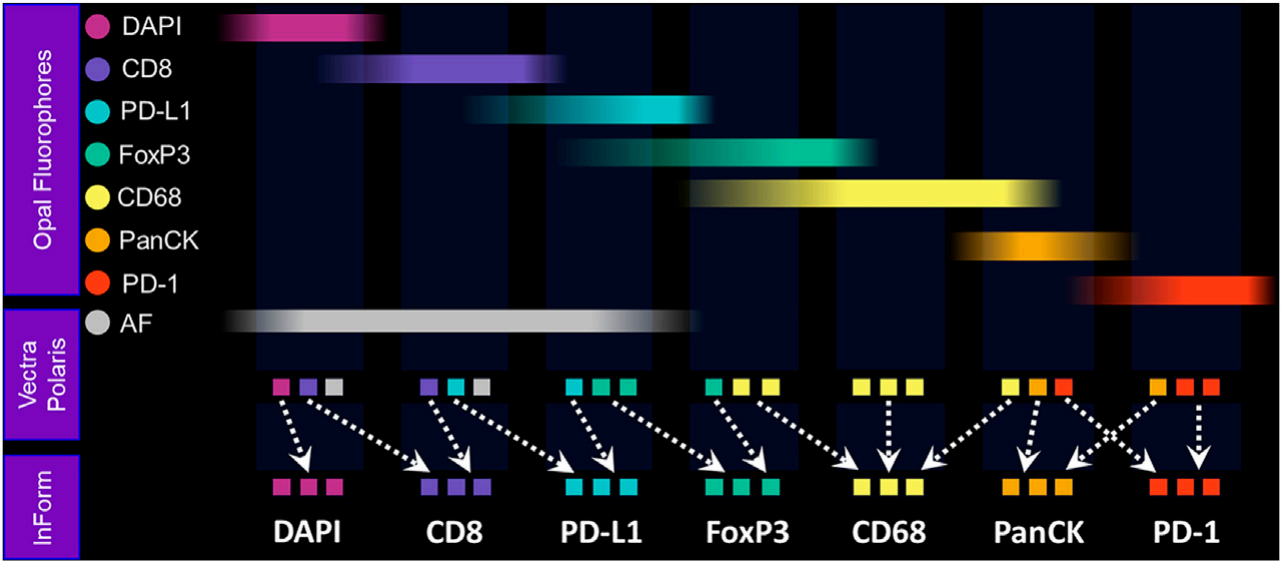
Fundamentals of MOTiF imaging and spectral unmixing. With the MOTiF workflow, a tissue is stained with Opal fluorophores using a Leica BOND RX autostainer. The 6-plex, 7-color assay is then imaged using the Vectra Polaris slide scan protocol, wherein whole-slide scan images can be acquired in 10 min. Using inForm, designated library slides are used to isolate the exact spectral signature of each fluorophore to properly unmix each whole-slide composite image, as well as isolate and remove tissue autofluorescence. Spectral unmixing of signals is key to the Phenoptics technology and critical to ensuring accurate data for analysis.

The Vectra Polaris was designed from the ground up to be an IF quantification system. Recent advances have been incorporated into the instrument to further support translational workflows, including a whole-slide multispectral imaging capability called MOTiF™, enabling the rapid 15-min scanning of 1.5 cm^2^ tissue areas for 6-plex, 7-color assays (Figure 4).

Following whole-slide image acquisition, images are analyzed with inForm image analysis software to quantify the cell-level biological features. The inForm software program was developed to integrate multispectral capabilities with image analysis to (1) spectrally unmix and isolate multiple Opal signals and background autofluorescence; (2) detect different tissue architecture (e.g., tumor, stroma, vessels, and necrosis) using a machine learning–based neural network pattern recognition function; (3) segment individual cells starting with nuclei, based on DAPI, and using other markers to detect membranous and cytoplasmic regions of cells; and (4) identify cell types of interest based on marker signal levels and cellular staining pattern using user-trained multinomial logistic regression algorithms.

To assist with image storage, sharing, and whole-slide image processing, a cloud-based platform called Proxima has been developed. Proxima is a hybrid solution consisting of a network-attached server (NAS) connected locally to the Vectra Polaris. Images generated on the Vectra Polaris are automatically transferred to the NAS and then uploaded to the cloud for remote viewing and data processing. The NAS can be used for rapid algorithm development and analysis of smaller projects, avoiding time delays associated with downloading images from cloud storage. Once image analysis algorithms are developed and validated locally, they can then be uploaded to Proxima for rapid batch whole-slide analysis, leveraging the computational power and speed of the cloud.

Analyzing whole-slide imagery generates very large data tables of single cell data, consisting of each individual cell’s classification according to the cell phenotyping function; all measured attributes, including signal levels in cellular compartments, staining pattern statistics, spatial coordinates, and tissue region designations; and any other spatial parameter established in the image analysis protocol. To reduce and consolidate these datasets into per-sample or per-slide statistics that can be used as bases for sample scoring, we have developed a library of open-source R-script packages, including phenoptr and phenoptrReports (akoyabio.github.io/phenoptr/; akoyabio. github.io/phenoptrReports/). These scores are often selected and optimized to quantitate the specific biological attributes, including spatial measurements, which correlate best with clinical parameters such as response to therapy.

### Assay Development Recommendations

In this section, we provide recommendations for assay development and optimization. Much of these insights were gained while developing and refining a rigorous assay optimization process and high-throughput slide analysis workflow in Akoya’s contract services laboratory. Additional detailed guidance can be found here: www.akoyabio.com/support/reagents/.

First, we suggest starting with validated IHC chromogenic assays for each of the markers. A validated assay, to us, refers to antibodies that have been tested using multiple titers and antigen retrieval conditions on control tissues to screen for markers which produce the best staining patterns. This usually includes cross-validation with other clones targeting the same epitope, with Western blots, and with a pathologist who is familiar with the target and can confirm the associated biology and staining pattern. It is also important that IHC assay be amplified to the point where the nonspecific background is on the verge of becoming apparent and interfering with the weakest specific staining. It is believed that at this point, the maximum sensitivity of the assay is achieved.

The next step in assay panel development is to design the multiplex panel by pairing Opal fluorophores with markers. It is recommended to pair the brightest Opal fluorophores with the weaker expressing proteins, and vice versa. More detailed information is available about Opal-marker pairing recommendations using the Assay Development Guide: www.akoyabio.com/support/reagents/.

**FIGURE 5:**
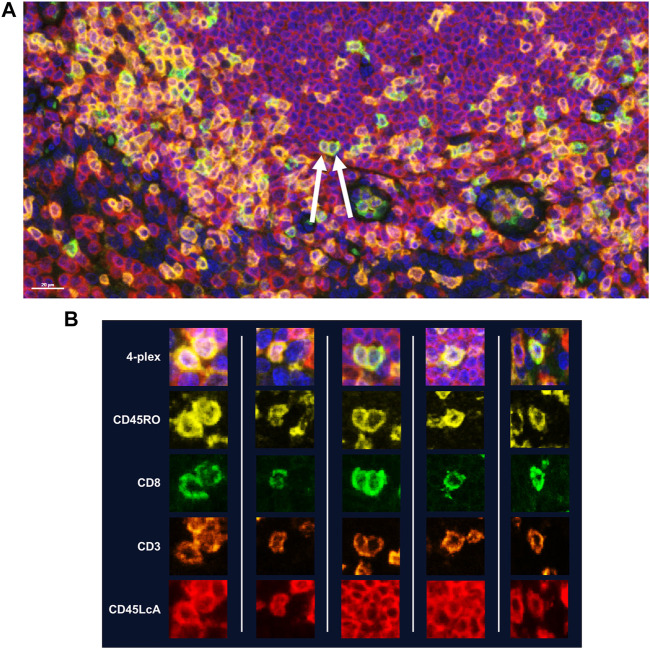
Assessment of TSA staining interference in detection of multiple markers within the same cellular compartment. Four membrane markers were chosen for this experiment which are known to have significant co-localization with one another: CD8 (Opal 520), CD3 (Opal 620), CD45RO (Opal 570), and CD45LcA (Opal 690). All reagents were double dispensed using the BOND RX and scanned using the Vectra Polaris. **(A)** Image with arrows indicates cells that display all four markers. **(B)** Additional representative images of cells displaying all four markers without any reduction in signal intensity demonstrating that TSA does not interfere with detection of three or more markers co-localized to the cell membrane.

While manual staining can achieve excellent results, it is recommended that autostainers be used to achieve quicker, consistent results. If using a BOND RX to perform the staining, double-dispensing primary antibodies, secondary antibody-HRPs, and Opal fluorophores are recommended. Double dispensing provides a more complete and uniform distribution of reagents across the tissue section, delivering uniform staining across large samples, regardless of where the section is mounted on the slide. Double dispensing of these reagents also appears to substantially eliminate “umbrella effect,” which is a term commonly used to describe when a previously applied marker impedes the application of an additional marker that co-localizes with the first. This is particularly important in instances where a user is interested in studying more than three markers of interest on the same cellular compartment. To demonstrate the effectiveness of the BOND RX double-dispense approach, an experiment was devised using CD3, CD8, CD45RO, and CD45LcA, membrane markers known to have significant co-localization with one another, to assess staining interference (Figure 5A). Results indicate negligible interference between the four markers, demonstrating reliable and clean detection of quad-positive cells, confirming that the “umbrella effect” is not an inherent limitation of TSA-based biomarker detection (Figure 5B).

It is recommended that one start with converting each IHC protocol to a monoplex IF (monoIF) protocol using the same primary antibody concentration established in the IHC assay and adjusting Opal TSA concentration to achieve fluorescence intensity signals at levels within suggested ranges. Reducing the primary antibody concentration should only be done in instances of where fluorescent signals continually remain high despite reducing the TSA concentrations, or if background staining becomes an issue.

To assess equivalence between chromogenic IHC and monoIF, we suggest rigorous image analysis to count positive cells and to confirm that the number of cells revealed with IF is equivalent to the number revealed by IHC, within a range of 10%–20%. Since one image type is of brightfield chromogenic staining and the other IF, each requires very different image analysis approaches.

If measured cell counts with the chromogenic IHC are significantly higher than that detected with IF, despite the fact that the fluorescent signals are within the recommended range, it may be helpful to try other secondary-HRP systems that come in a more concentrated form than the current Akoya commercially available secondary HRP system. Opal users find that products such as Powervision from Leica increase the signal from lower expressing cells while not overly amplifying signal from stronger expressing cells. If the signals for higher expressing cells become too bright beyond recommended levels, reducing the primary antibody concentration can return the fluorescent signals back into the recommended ranges.

Increasing the signal of lower expressing cells while not significantly increasing the signal from higher expressing cells suggests that there is some level of saturation occurring. The tradeoff between dynamic range and sensitivity should be considered when optimizing an assay. Is it more important to see every low expression cell or to maximize dynamic range? In our experience, using a more sensitive secondary detection system to reveal low expressors retains at least two orders of magnitude of signal to resolve low, medium, or high expression levels when important to the biomarker assay.

**FIGURE 6:**
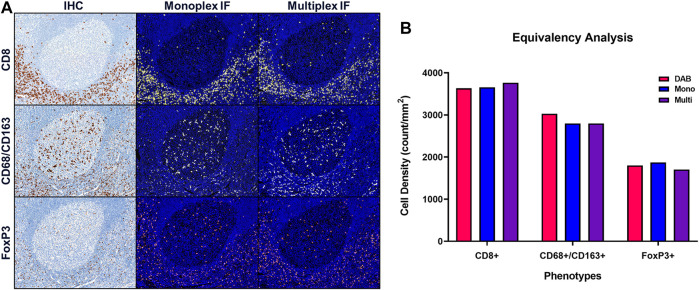
Quantitative assessment of equivalence to chromogenic IHC staining. Staining parameters for each antibody are first optimized using single stain, chromogenic IHC on tonsil sections. Next, each primary antibody was paired to a select TSA fluorophore and a single-stain, monoplex IF (monoIF) was performed. Lastly, all of the monoplex conditions are combined into a multiplex IF (mIF) panel. **(A)** Representative image showing serial sections with each marker for IHC, monoIF, and mIF staining. **(B)** Quantitative assessment of each image demonstrating the equivalence of staining for each marker across the three staining parameters, wherein monoIF and mIF are consistent with DAB.

Once equivalence between chromogenic IHC and monoIF is achieved, the monoIF protocols are then combined into a multiplex IF (mIF) protocol. The equivalency test, illustrated in Figure 6A, consists of a 15-slide serialization as described and is an efficient approach for evaluating IHC/monoIF/mIF equivalence (Figure 6B).

**FIGURE 7:**
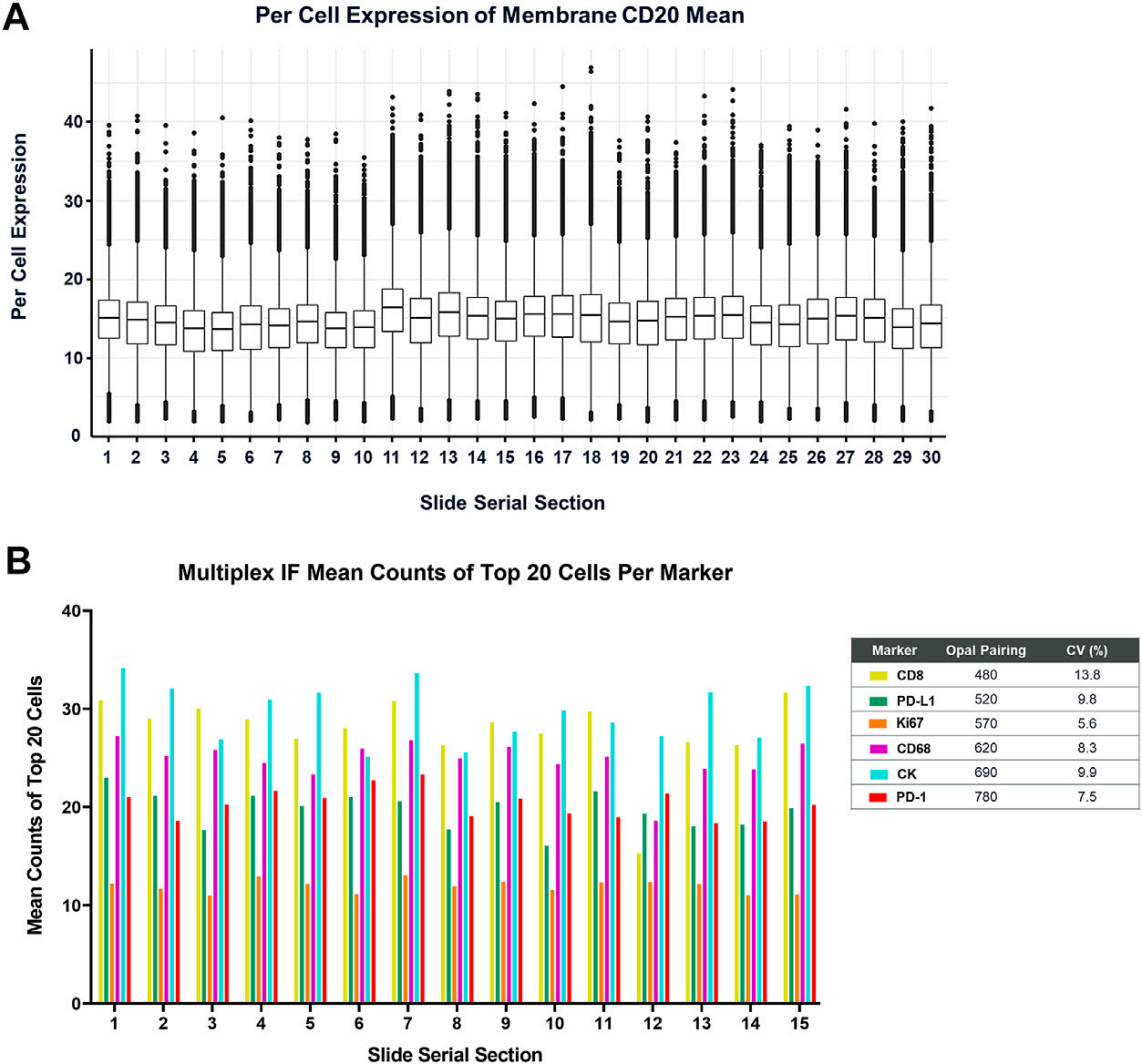
Measurement of the BOND RX performance is captured by **(A)** running a CD20-Opal 520 monoplex assay across 30 serial sections. The percent coefficient of variation (%CV) is calculated by determining the mean expression of the top 20 brightest expressing cells across five matching annotations on all the serial sections. **(B)** Similarly, an optimized 6-plex, 7-color assay is also run on 15 serial sections, and the mean IF counts of the top 20 cells per marker are measured and the mean, standard deviation, and %CV are calculated.

Lastly, routine maintenance and regular performance testing of the BOND RX is critical for obtaining consistent and reliable data. A basic challenge of any IHC or IF assay is distinguishing true negative staining from staining failures. In Akoya’s Contract Research Services division, we perform monthly performance tests on every BOND RX instrument, consisting of one batch of 30 tonsil serial sections stained with a monoIF protocol labeling CD20 (Figure 7A) and a second batch of 10 tonsil serial sections that are stained with a standard PD-1/PD-L1 6-plex, 7-color mIF protocol (Figure 7B).

To gauge staining performance, we carefully select a minimum of five fields of view that are aligned across serial sections to reduce the impact of tissue heterogeneity on the staining reproducibility measurement. The stain intensity for each slide is then determined as the average of the top 20 brightest cells. When instruments are well maintained and a high-quality tissue control is used, such as healthy tonsil, percent CVs should be in the 5%–15% range. When using this methodology, it is important to always inspect the imagery as well because either approach cannot detect issues related to staining artifacts such as folds or other staining errors.

### Characteristics of an Optimized Assay

The key performance parameters of a well-optimized assay are signal strength, signal balance, marker independence, staining uniformity, reproducibility over time, and most importantly from a translational perspective, the ability to transfer assays across sites with equivalent results.

#### Signal Intensity

As measured by inForm or Phenochart software, the target range for positively stained pixels is in the 10–30 normalized count range for all Opal fluorophores, with the exception of Opal Polaris 780, where the recommended range is from 1 to 10 cell counts. These ranges support reliable and accurate data analysis. It is worth noting that viable data can still be obtained when signals are as low as a few counts or as high as 50 or more counts, but risks are higher for crosstalk issues.

#### Dynamic Range

Our standard approach assessing dynamic range is to calculate a signal-to-background (SNR) ratio by dividing the average of the top 20 brightest cells by the average intensity of the weakest 10% of cells. An SNR of 10 or more supports reliable image analysis, including accurate counting of positive cells and quantifying expression levels. While we recommend an SNR of 10 or greater, typical ratios are well in the 100s with high-performing antibodies, or as low as 3-to-1 that still provide analytical value.

#### Signal Balance

With the classic Opal line-up (Opals 520, 540, 570, 620, 650, and 690), the rule of thumb was to aim for ratios of signals between neighboring channels of 3:1 or less. This rule was particularly useful for the 520, 540, and 570 channels. This was just a guide. Most of the time when ratios exceeded 3, the assays performed very well with negligible crosstalk.

With the introduction of MOTiF 6-plex, 7-color capability, which replaces Opal 540 with Opal Polaris 480 and Opal 650 with Opal Polaris 780, the rule of thumb substantially goes away because the six fluorophores are more spectrally distinct. As a result, there is little residual crosstalk after unmixing, even if neighboring signals are significantly imbalanced. As discussed in the signal intensity section, normalized counts within the 10–30 range for all Opals except Opal Polaris 780 are key to achieving optimal signal balance and an SNR of 10 or more. In the end, the goal of signal balancing is to achieve negligible crosstalk.

#### Crosstalk

Crosstalk should be minimized or eliminated because it can cause false positives and can limit dynamic range for important expression markers when crosstalk inaccurately contributes to a neighboring signal channel. There are two main sources of crosstalk: (1) instrumental crosstalk occurring when fluorescence signals leak from one channel to another due to imperfect filter optics or from inadequate crosstalk compensation algorithms and (2) staining crosstalk from actual fluorophore inaccurately labeling proteins on the sample, resulting in residual fluorophores inadvertently binding to epitopes intended to be labeled by other fluorophore. It is very important to distinguish the two causes because resolving each is a very different process.

When optimizing a multiplex assay, visual assessment for spectral bleed-through should always be part of the evaluation process because trained human perception is very good at distinguishing actual signal from crosstalk.

Crosstalk can be assessed with a set of monoIF slides, one for each Opal fluor. It is then determined by dividing the signal in its respective channel from an image of the monoIF sample corresponding to that channel by the signal in that channel from an image of a sample that is only stained with the neighboring fluorophore. For a robust assay, residual crosstalk of less than 1% is recommended to ensure minimal interference with image analysis. More often than not, there is no measurable crosstalk.

Specific guidance on this topic is provided in our guide available on the Akoya website: https://www.akoyabio.com/support/reagents/.

#### Reproducibility

**FIGURE 8:**
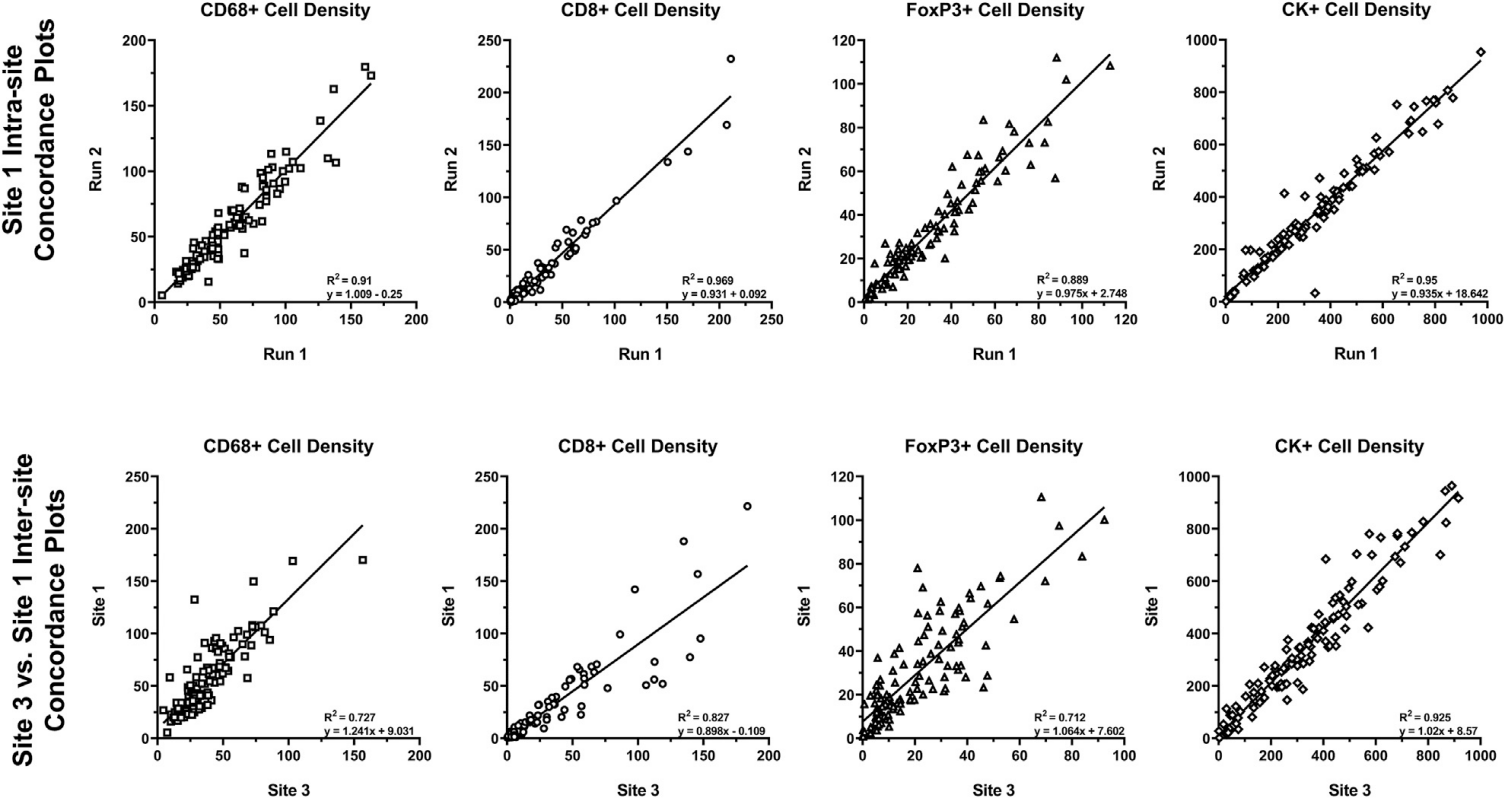
Intra- and inter-site concordance assessment of cell densities. Average intra- and inter-site concordance plots comparing Run 1 vs. Run 2 (intra-site) and Site 1 vs. Site 3 (inter-site) cell densities for CD68^+^, CD8^+^, FoxP3+, and CK+. Data shown as R^2^ (slope and standard deviation (SD) of slope). Graphs depict data presented as part of a study investigating multiplex IF reproducibility across multiple institutions (Hoyt et al., 2019).

Reproducibility of approximately 10% CV or better is typical of well-optimized panels and run on the Leica BOND RX that is well maintained. To assess the analytical performance of multispectral mIF and its suitability to support future clinical applications, the MITRE study was conducted, as previously discussed (Hoyt et al., 2019). Serial sections of tonsil and tissue microarrays and reagent kits were distributed to six sites, each equipped with a Leica BOND RX and a Vectra Polaris. Slides were stained with an optimized assay panel for PD-1, PD-L1, CD8, CD68, Foxp3, and CK using the recommendations described. Intra- and inter-site concordance analysis of signal intensities was assessed (Figure 8).

Comparison of the multispectral mIF and IHC cell counts showed equivalence of 90% on average. Intra-site equivalence assessment showed an average slope of 0.93 and R-squared value of 0.86. Inter-site assessment showed a slope of 0.98 and R-squared value of 0.76, confirming analytical robustness.

Beyond demonstrating that the staining was reproducible across sites, we were able to establish that image analysis substantially addresses the inconsistencies of human visual assessments. Agreement among sites for assessing percent positivity of PD-L1 in immune cells, using the TMA samples, was demonstrated by an R-squared value of 0.81 and slope of 0.82. In contrast, ICC values of <0.3 were demonstrated for a similar assessment of reproducibility in the NCCN and Blueprint 2 PD-L1 IHC harmonization studies (Hirsch et al., 2017; Rimm et al., 2017).

#### Photostability

To assure analytical robustness and support eventual clinical applications, it is important that fluorescence signals are not only photostable to allow for repeated scanning but also temporally stable so that slides can be stored for months without appreciable loss of signal. Multiplex assays using Opal TSA detection can be scanned repeatedly over the course of 6 months while being stored at room temperature with <10% loss of signal. In an internal assessment, we determined that signal intensity across repeated scanning decreased linearly by <6% over 30 scans. This consistent level of photostability is due to the nature of TSA-based labeling that involves covalent binding of fluorophores to tyrosine residues, in addition to the pulsed LED excitation in the Vectra Polaris imaging instrument, which illuminates the sample only during imaging, reducing total light exposer by many factors of magnitude compared to conventional fluorescence microscope excitation systems.

## Translating Multispectral mIF Into the Clinic

With well over 100 peer-reviewed publications that utilize multispectral mIF, the I/O research community has embraced mIF as a primary tool to uncover and characterize cell-level biological interactions in the TME, to help understand how cancer survives and grows, and to uncover its potential biological mechanisms to target with new therapies. Moreover, multispectral mIF has become the leading candidate to support identification of urgently needed predictive biomarkers to make I/O more efficient and precise. As the predictive power of biomarkers based on the spatial biology is revealed by mIF methods, we fully expect mIF to translate into clinical practice as an essential tool in a physician’s diagnostic toolkit.

The purpose of this final section is to suggest remaining steps to translate multispectral mIF into a fully validated clinical platform. Requirements can be summarized into five categories: (1) flexibility to fully explore co-expression and spatial information; (2) analytical performance providing reproducibility and robustness; (3) workflow and standardization to support laboratory needs; (4) demonstrated clinical validation and utility; and (5) reimbursement from payers to support laboratory economics and clinical adoption.

### Flexibility to Fully Explore Co-Expression and Spatial Information

The academic and medical research setting where oncologists, pathologists, immunologists, cancer biologists, and image analysis scientists work together to solve challenging life science problems is ideal for exploring the full dimensionality of spatial biology. Supporting these interdisciplinary teams in their pursuit of effective biomarkers requires a research platform that is open and flexible and that fits into research budgets and laboratory workflows. Open and flexible in this context refer to the ability to freely select antibodies, design multiplex panels, adjust amplification to capture expression levels that correlate best with clinical parameters, and follow the data to explore the intricate biology behind I/O responsiveness.

The objective of I/O research is to quickly converge on optimum biomarker signatures, which typically means integrating (1) hypothesis-driven sets of markers; (2) staining protocols, including optimized antigen retrieval and amplification to observe the range of expression related to response; (3) image analysis measurements of co-expressions and spatial parameters; and (4) calculations or algorithms to reduce large cellular datasets to operator-independent and actionable scores, which we define as scores based on measurements of TME cell-level biology that have sufficient utility to justify use in making therapeutic decisions. The platform should have the flexibility to freely adjust parameters for each of these steps, allowing researchers to effectively integrate a translational workflow into their daily routine and fully explore the spatial biology to identify optimum predictive biomarkers.

### Analytical Performance Providing Reproducibility and Robustness

Analytical performance provides confidence that assay results are accurate, regardless of when, where, or by whom the assay is performed. Performance standards need to be at a level typified by, at a minimum, the regulatory analytical standards of a laboratory in compliance with the US Clinical Laboratory Improvement Amendments (CLIA) and, ideally, the analytical component of a Food and Drug Administration *In Vitro* Diagnostic (IVD) Class III Medical Device Pre-Market Approval submission. Components of these standards include (1) precision and reproducibility (e.g., CV) of the test readout over time, across instruments, across operators, across sites, and so on; (2) shelf life and stability; and (3) robustness, which is a measure of a platform’s capacity to remain unaffected by small but deliberate variations in method parameters.

Analytical performance applies to image analysis as well, but in a different way. Image analysis algorithms will provide the same answer every time for a given sample, but it can be challenging to provide accurate data due to the variability of staining, tissue morphology, and tissue conditions. Accuracy across sample variability needs to be assessed, preferably using a pathologist’s manual assessments and/or annotations as a gold standard.

Although there has yet to be an IVD-level validation of an mIF assay, results from the MITRE study described above suggest that multispectral mIF has the performance attributes suitable to support the analytical requirements of an FDA-approved IVD.

### Workflow and Standardization to Support Laboratory Needs

As mIF matures and moves toward the clinic, there is a push to define standards for developing and validating predictive biomarkers, including multispectral mIF. In 2017, the National Institutes of Health launched a $220 million initiative called the Partnership for Accelerating Cancer Therapies (PACT) in which drug companies facilitated systematic and uniform clinical testing of biomarker assays (https://fnih.org/what-we-do/programs/partnership-for-accelerating-cancer-therapies). The Society for Immunotherapy of Cancer (SITC) launched a benchmark effort of its own in 2019, establishing a 21-member task force to develop best practices surrounding the use of multiplex IHC and additional multiplex imaging tools (https://www.sitcancer.org/membership/volunteer/task-forces/pathology).

Platform providers meanwhile will need to design assays that are robust across the variability of human tissue specimens, incorporate suitable controls to compensate for staining variations, automate and integrate components of the assay to reduce the likelihood of errors, and create levels of access to assure platform configurations are controlled and locked down.

Once these items are accomplished, the next critical step is to provide the platform with a configuration that satisfies the needs of the clinical laboratory setting. Processing a sample must be reduced to a simple, streamlined workflow that resembles, as much as possible, an automated sample-in, score-out process. Given the complexity of mIF or IHC, the measurement, and the variability of tissues, there will be a few points during the process that will need pathologist input, including quality assessment, tumor annotation, and results review. Since the platform performance depends on the proper execution of each step and as the success of subsequent steps depends heavily on the performance of previous steps, the entire end-to-end workflow needs to be automated and locked down to prevent operator dependencies. Some recommendations to improve performance and reduce assay variability at each step include full integration into a laboratory information management system to automate information management and avoid errors by using a database to indicate autostainer protocol, confirm appropriate reagents, select image acquisition protocols (exposures, colors, sequence, etc.), and develop image analysis and reporting algorithms.

This workflow also needs to support laboratory staff who operate the instruments and pathologists who provide valuable quality control and oversight function to confirm the sample is sufficient for testing, and to review and approve results in the form of a report.

Providing an H&E view of the sample will be critical for the pathologist’s tissue quality inspection, annotation of tumor, and results review. mIF imagery, while visually stunning, is foreign to most classically trained pathologists and does not present the anatomical and morphological features in a format that most pathologists are accustomed to. Ideally, the H&E view will be of the exact same section that is analyzed with multispectral mIF, rather than of another section from the biopsy sample. Although there will probably be a representative H&E-stained section from each sample tissue block, the representative H&E section may be from a very different depth into the block and may contain significantly different tissue morphology, thus not providing sufficient visual guidance about the makeup and quality of the section being characterized with mIF. Additionally, as the reference H&E slide is a different section, it may have different sectioning artifacts such as tears, folds, and lost areas. To address this issue, we have incorporated into our workflow a method to capture an H&E whole-slide image of the section to be stained and analyzed with multispectral mIF. The H&E view and the appropriate representations of the multispectral mIF views will be used by the overseeing pathologist as part of his or her review of the results and final sign-off of a report.

Other basic requirements for translation are that (a) instruments, reagents, and software are designed and manufactured within an ISO13485-certified quality system and according to good manufacturing principles, typically audited by the FDA for Class-III medical devices; (2) the platform workflow is compatible with common and custom laboratory information management systems; and (3) data processing workflows support remote viewing and annotation and are capable of handling the scale and size of images and datasets, probably requiring a cloud-based platform that is HIPAA compliant. A key attribute of a cloud-based solution is that the computational power supports rapid automated whole-slide image analysis taking on average 10 min per slide, which will be needed to provide sufficient turnaround time and reduce the massive amount of raw data for each tissue section to an operator-independent and actionable score.

### Clinical Validation and Utility

Potentially, the most important element of translating these methods to clinical practice is demonstrating analytical validation, clinical validation, and clinical utility in the clinical trial setting. Having a platform and assays that support the rigors and quality and regulatory requirements of clinical trials, coupled with clinical-grade analytical performance, are critical. Additionally, laboratories running the trials need to have appropriate documentation and controls in place, as well as be CLIA/GCP/GCLP certified or compliant. They also need to have daily slide analysis throughput to support trial timelines.

#### Clinical Test Reimbursement to Support Laboratory Economics and Clinical Adoption

Reimbursement is also a key milestone in the path to clinical adoption, as important as demonstrating clinical validation and utility. Despite significant attention being paid to personalized (i.e., precision) medicine, there is still significant pressure to reduce testing costs. Obtaining reimbursement is a complicated process and requires significant time and resources to demonstrate rigorously real value. Regulatory and reimbursement bodies, such as CMS and NCCN, have made the hurdles higher because of the lack of performance of many over-sold testing platforms. On the other hand, there are several examples of tests, such as next-gen sequencing–based tests for microsatellite instability and tumor mutation burden, that are garnering healthy reimbursement and that have predictive power at levels that will be potentially superseded by assays based on mIF assessments.

Obtaining approvals and support for reimbursement from regulatory agencies requires clinical utility studies that demonstrate significant statistical evidence that patients and the health system benefit from taking the test. An example of a clinical utility study that effectively demonstrates the clinical utility of a multispectral mIF was performed by Peabody et al. for a prostate cancer prognostic test (Peabody et al., 2017).

Lastly, the cost of performing the test must fit within the economic imperatives of academic and commercial reference laboratories. Simply put, the initial investment, per-test cost, and volume need to support healthy business for the laboratory, certainly enough to cover costs, but optimally to support healthy gross and profit margins.

## Conclusion–Multiplex Immunofluorescence Has a Bright Future

In the ongoing battle against cancer, there are two major developments that give us reason to be optimistic about improving the lives of cancer patients. First, I/O has drastically changed the game and created an extensive list of new efficacious avenues of attack by harnessing the immune system and enabling new drug combinations that work synergistically together. Second, thanks to new detection and imaging technologies, our understanding of the TME and cancer immunology is advancing at a rapid pace, revealing driver biology behind progression and responses to therapy.

In addition to the multispectral mIF approach described here, there are many other new and higher-plex discovery platforms taking root in cancer research (Tan et al., 2020; Taube et al., 2020). These platforms leverage novel detection approaches to multiplex tens of proteins in single tissue section, such as cycled steps of staining and imaging, faster scanning of laser or ion beams coupled to mass spectrometers to analyze antibody-metal atom conjugates, spatially indexed beads for imaging “trans-scriptomics,” and spatially resolved oligo-barcoded snipping technologies to look at proteins and RNA in optically masked areas. These approaches give researchers a comprehensive tool kit to explore and understand the intricate details of how cells behave in the TME.

Fortunately, biomarker discoveries made with these higher-plex approaches, which have workflows and economics that are not well suited to translation into the clinic, can be reduced to the most informative markers in a multispectral mIF workflow providing rapid, whole-slide analysis that is automated and operator-independent for trials and clinical deployment. They provide a rich pipeline of new biomarker signatures that can be converted to a multispectral mIF assay which is suitable for clinical trials and translation into eventual standard of care. The new frontier of biomarker discovery based on spatial biology has a practical path toward the clinic, based on practically, economically, and analytically robust workflows, which promises to have material benefit for cancer patients.
